# Supplementary material for: Cholinergic activation of HLH-30/TFEB promotes C. elegans infection resilience
Source: bioRxiv. 2026 Sep 24:2026.09.18.752639. Preprint. [Version 1] doi: 10.64898/2026.09.18.752639 (PMC13622048; doi:10.64898/2026.09.18.752639)
Supplement: Supplementary file 1 [file NIHPP2026.09.18.752639v1-supplement-1.pdf]

bioRxiv preprint doi: https://doi.org/10.64898/2026.09.18.752639; this version posted September 24, 2026. The copyright holder for this preprint (which was not certified by peer review) is the author/funder, who has granted bioRxiv a license to display the preprint in perpetuity. It is made available under a CC-BY 4.0 International license.

Supporting information

S1 Data. Supporting numerical values. Numerical values underlying the 18 graphed panels of Figs 1 to 6, one sheet per panel, each giving the tabulated replicate values, the summary statistics, and the statistical test reported in the corresponding figure legend. Sheets Fig5B_AllTrials and Fig6C_AllTrials also give the survival data and the log-rank statistics for every independent trial. Sheet RT-qPCR Primers gives the primer sequences used for RT-qPCR. Sheet Index lists the contents. (XLSX)
